# 

**Published:** 1867

**Authors:** 


### CONTENTS FOR APRIL AND MAY.

ORIGINAL CONTRIBUTIONS.
A Case of Complete Prolapsus of the Rectum, operated upon by "Smith's Method." By J. W. Freer, M.D., Chicago, ... 145
A New Form of Suture. By Albert H. Hoy, M.D., Racine, Wis., ... 150
Fracture of Astragalus.—Dislocation of Foot Inwards. By Henry T. Godfrey, M.D., Benton, Wis., ... 252
Clinical Lectures. By Wm. C. Lyman. M.D., Chicago, ... 153
Typhlitis. By A. T. Hudson, M.D., Lyons, Iowa, ... 159
A Case of Puerperal Convulsions.—Additional Testimony in Favor of Belladonna. By J. Ramsey Flood, M.D., Detroit, Mich., ... 161
Milk-Sickness and the Illinois Legislature. By W. Anderson, M.D., Pontiac, Ill., ... 163
A Case of Puerperal Convulsions Effectually Controlled by the Inhalation of Chloroform. By John W. Hensley, M.D , Yates City, Knox, Co., ... 155
Scirrhus of the Liver and Stomach, Tuberculosis, and Partial Fatty Degeneration of the Kidneys. Hospital Record, ... 166
Phthisis Pulmonalis as a Sequel to Suppressed Menstruation, ... 171
Veratrum Viride, ... 173

PROCEEDINGS OF MEDICAL SOCIETIES.—Fox River Valley Med. Assoc. ... 174
Pulaski County (Ind.) Medical Society, ... 175
Kalamazoo Valley, (Mich.,) Medical Association, ... 176
The Endoscope, and its Application to the Diagnosis and Treatment of Urinary Affections. By A. J. Desormeaux, M.D., Translated by R. P. Hunt, M.D., ... 177

EDITORIAL.—Double Number ... 209
Exchanges and Correspondents, 209
Rush Med. Col.—New Building, 210
Practical Anatomy, ... 213
The Chicago Board of Health, ... 217
Criminal Abortion, ... 221
Cincinnati Again, ... 223
Pass Him Around, ... 224
"Why Not?" ... 255
Copyright, ... 225
Wants to Know, ... 225
Books Received, ... 226

LOOT.—Cholera, ... 228
Vesical Calculus, ... 228
Clitoridectomy, ... 228
Poisoning by Strychnine, ... 229
Milk-Sickness ... 229
Elimination in Cholera, ... 229
Hydrophobia, ... 231
Dry Tongue, ... 231
Light for the Endoscope, ... 231
Apropos of the Endoscope, ... 232
Belladonna—Scarlatina, ... 232

NEWS AND ITEMS.—Dr. Storer, ... 232
To Physicians, ... 232
Resignation of Prof. Gunn, ... 233
Comp. Letter to Dr. Lewitt, ... 233
Prof. M. Gunn, ... 234
Dr. Lewitt, ... 234
Southern Jour. of Med. Science, 234
New Medical Journal ... 234
University of Michigan ... 235
Medical Bookstore, ... 235
Meeting of the Societies, ... 235
Int. Med. Congress at Paris, ... 235
The Int. Medical Congress, ... 236
Illinois State Medical Society, ... 236
Indiana State Med. Society, ... 236
Com. on Prac. Med. & Epidem ..., 237
Erratum, ... 237
Married, ... 237
Obituary ... 238
Back Nos. of Journal Wanted, ... 238
Advertisement, ... 238
Receipts Acknowledged, ... 238
Mortality Report for February, ... 239
Mortality Report for March, ... 240

